# Subsequent intra-abdominal fibromatosis mimicking recurrent gastrointestinal stromal tumor

**DOI:** 10.1186/1746-1596-8-125

**Published:** 2013-07-31

**Authors:** Dongxian Jiang, Deming He, Yingyong Hou, Weiqi Lu, Yuan Shi, Qin Hu, Shaohua Lu, Chen Xu, Yalan Liu, Ju Liu, Yunshan Tan, Xiongzeng Zhu

**Affiliations:** 1 Department of Pathology, Zhongshan Hospital, Fudan University, Shanghai 200032, P.R. China; 2 Department of Oncology Surgery, Zhongshan Hospital, Fudan University, Shanghai 200032, P.R. China; 3 Department of Pathology, Cancer hospital, Fudan University, Shanghai 200032, P.R. China

**Keywords:** GIST, Intra-abdominal fibromatosis (IAF), Imatinib

## Abstract

**Abstract:**

Intra-abdominal fibromatosis (IAF) commonly develops in patients who had abdominal surgery. In rare instances, it occurs subsequent to gastrointestinal stromal tumor (GIST). This special situation has clinical significance in imatinib era. About 1000 patients with GIST in our institution from 1993 to 2010 were re-evaluated based on their clinical and pathological data, the treatment strategies and the follow-up information. We identified 2 patients who developed IAF after GIST resection. Patient 1 was a 54 year-old male and had 5 cm × 4.5 cm × 3.5 cm jejunal GIST excised on February 22, 1994. Three years later, an abdominal mass with 7 cm × 6 cm × 3 cm was identified. He was diagnosed as recurrent GIST from clinical point of view. After excision, the second tumor was confirmed to be IAF. Patient 2 was a 45-year-old male and had 6 cm × 4 cm × 3 cm duodenal GIST excised on August 19, 2008. One year later, a 4 cm mass was found at the original surgical site. The patient refused to take imatinib until the tumor increased to 8 cm six months later. The tumor continued to increase after 6 months’ imatinib therapy, decision of surgical resection was made by multidisciplinary team. The second tumor was confirmed to be IAF with size of 17 cm × 13 cm × 11 cm. Although IAF subsequent to GIST is very rare, it is of clinical significance in imatinib era as an influencing factor for making clinical decision.

**Virtual slides:**

The virtual slide(s) for this article can be found here: http://www.diagnosticpathology.diagnomx.eu/vs/1076715989961803

## Introduction

Gastrointestinal stromal tumor (GIST) is the most common gastrointestinal mesenchymal tumor and mainly treated with surgical resection in the past era without effective drugs. The identification of *KIT* mutations in recent years has led to the development of specific, targeted therapies with tyrosine kinase inhibitors such as imatinib mesylate (STI571, Gleevec; Novartis Pharmaceuticals, Basel, Switzerland) and sunitinib malate (SU11248, Sutent; Pfizer, Inc, New York, USA), which are more effective for unresectable, metastatic and recurrent diseases [1].

With the accumulation of knowledges on GIST and long-time follow-up information, GIST patients are found to simultaneously or metachronously have other types of tumors [2-7], some of which are easier to be differentiated from GIST [4,6], while others might be confused with recurrent GIST from the clinical point of view [2,3].

Intra-abdominal fibromatosis (IAF) is a rare mesenchymal tumor which does not metastasize but tends to exhibit a high degree of local infiltration and invasion, thus becoming lethal in some cases [8-11]. IAF developed after abdominal surgery and individual with both GIST and IAF were reported recently [2,3] and non-random association between GIST and IAF was established very recently based on data from 10 medical centers [12].

In this report, we describe 2 additional GIST patients who admitted to our institution before and after imatinib era, respectively, and developed IAF at the site of GIST resection beds. Both of the two cases created the first impression of GIST recurrence. Surgical excision of the lesion was done without any hesitation in the first patient, however, difficult decision owing to the suspicion of metastatic disease when imatinib therapy was available in the second patient. These 2 cases highlight the importance of recognizing the coexistence of other diseases in patients with chronic GIST since the metachronous tumor subsequent to GIST is easy to be mis-regarded as recurrent tumor and treated with imatinib in molecular-targeted therapeutic era.

## Materials and methods

### Tumor specimens

Medical records and tissue specimens of about 1375 primary mesenchymal tumors of GI tract with the years ranging from 1993 to 2010 were retrieved from Zhongshan Hospital, Fudan University. Among them, 1055 cases were primary mesenchymal tumors previously characterized as leiomyoma, leiomyosarcoma, leiomyoblastoma, schwannoma, stromal or smooth muscle tumors originated from GI tract. Out of these 1055 cases, 997 cases underwent surgery and immunohistochemically or histologically identified as GISTs based on KIT positive immunohistochemical detection or histopathological spectrum with KIT-positive tissues. All tumor slides were reviewed by two experienced pathologists. Another 195 GIST patients were collected from our own consultant files from January 2003 to March 2010. Tumor tissue collection and the following analyses were approved by the review boards of Zhongshan Hospital, Fudan University.

### Clinical records

Patient demographics and clinical data were retrieved from the medical records. Data on gender, age at diagnosis, *KIT, PDGFRA* mutation status and follow-up information were collected.

### Histological evaluation

Hematoxylin and eosin (H&E)-stained slides for each patient were reviewed and the following features were recorded: predominant cell type, pleomorphism, nuclear atypia, necrosis, mitotic count, invasion, and risk levels [13].

### Immunohistochemical evaluation

Immunohistochemical staining was performed based on the method previously reported [14]. Formalin-fixed paraffin sections were prepared from one representative block and subjected to immunohistochemical staining with a panel of antibodies against CD117 (rabbit polyclonal anti-human KIT, diluted 1:150; Dako, Denmark), CD34 (mouse monoclonal antibody, clone QBEnd 10, diluted 1:200; Dako), α-smooth muscle actin (mouse monoclonal antibody, clone 1A4, diluted 1:200; Dako), desmin (mouse monoclonal antibody, clone D33, diluted 1:200; Dako), S-100 protein (polyclonal, diluted 1:300; Dako) and vimentin (mouse monoclone antibody, V9, diluted 1:1000; Dako). The slides were first treated with 0.01M citrate buffer (pH 6.0) by microwave method for antigen retrieval, and incubated overnight at 4°C. Immunohistochemical detection was performed with EnVision-based system using a commercial kit (Dako). Diaminobenzidine was used as the chromogen, and all sides were counterstained with hematoxylin.

## Results

### Report of two cases

**Figure 1:**
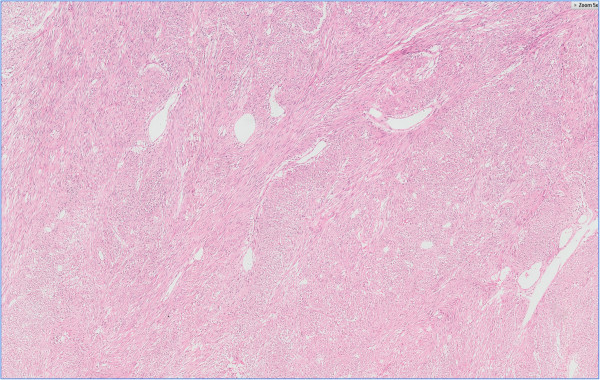
Characteristics of GIST showed spindle cells with intersecting growth pattern Hemotoxylin eosin staining 5 ×.

**Figure 2:**
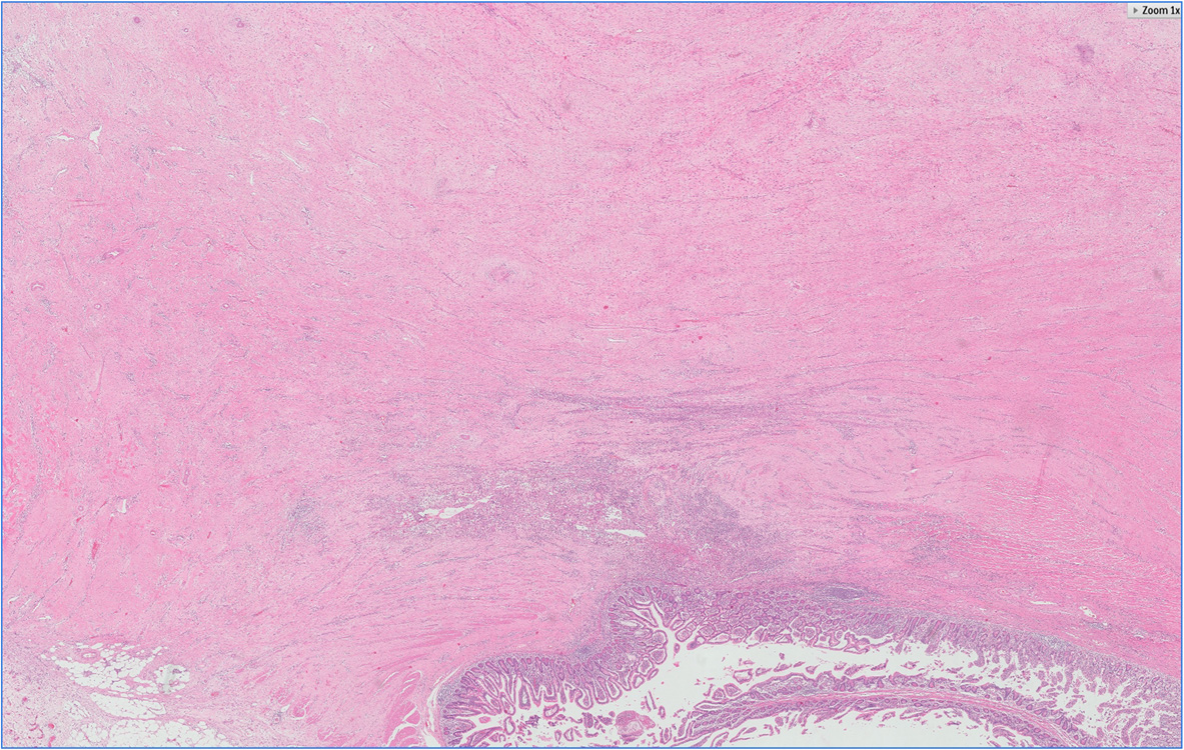
**Histological images (1 ×) of the tumor.** Hematoxylin-eosin staining showing 1) proliferating non-dysplastic fibroblasts, 2) prominent dilated thin-walled veins, 3) interwoven spindle cells and varying amounts of collagen, 4) tumor cells invading the muscle layer of the jejunum with intact mucosal layer.

Patient 1: A 54-year-old male admitted to Zhongshan Hospital (Fudan University, Shanghai, China) on Feb 8th, 1994 due to tarry black stools for four months. Gastroscopy found no abdominal changes. Mesenteric artery angiography showed a jejunal tumor. Emission computerized tomography (ECT) examination found active bleeding near the junction of the jejunum and ileum. Primary impression was small intestinal leiomyoma. The patient was treated with radical resection of part of the jejunum. Pathological examination found a non-mitotic submucosae tumor with a small ulcer. The tumor was 5 cm × 4.5 cm × 3.5 cm in size and showed outward growth pattern. The primary diagnosis was cellular leiomyoma and now reviewed as GIST based on morphology (Figure 1) and immunohistochemical staining results, which showed that the tumor cells were positive for CD117, but negative for CD34, a-SMA, desmin and S-100. The patient was classified into low risk level according to the current risk level classification [15]. He recovered smoothly after surgery and was disease free without any further therapy. However, in Feb 1997, he admitted to our hospital again due to back ache. Computed tomography (CT) scanning showed a mass in the retroperitoneum of the right lower quadrant. Since clinical impression was a recurrent tumor, he was treated with laparotomy without any hesitation. A lower right retroperitoneal tumor with partial small intestine and partial ureter were successfully excised. Gross examination found a 7 cm × 6 cm × 3 cm mass attached to small intestine, and the ureter was entrapped in it. The tumor sections were gray-white. Microscopical examination found spindle cells with abundant collagen, no demarcated boundary between the tumor cells and the muscularis propria of jejunum (Figure 2), and no more than 3 mitotic ureter cells per 50 high power fields. The tumor was different from the primary tumor morphologically. The tumor cells were negative for α-SMA, S-100, desmin, CD34 and CD117. He was diagnosed to have IAF after discussion with senior pathologists in Department of Pathology, Cancer Hospital, Fudan university since the situation was very rare in clinical practice. The patient survived another 18.5 years after two operations.

**Figure 3:**
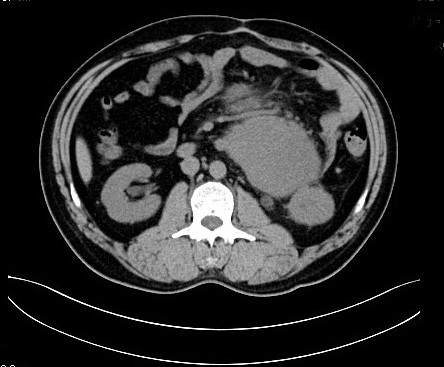
**Preoperative CT scan images of the tumor.** A contrast-enhanced CT image showing a homogeneous low-dense tumor with slight enhancement involving the proximal jejunum and descending colon.

**Figure 4:**
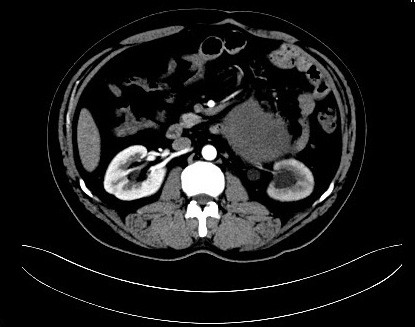
The upper ureter entrapped in the inside of the tumor.

**Figure 5:**
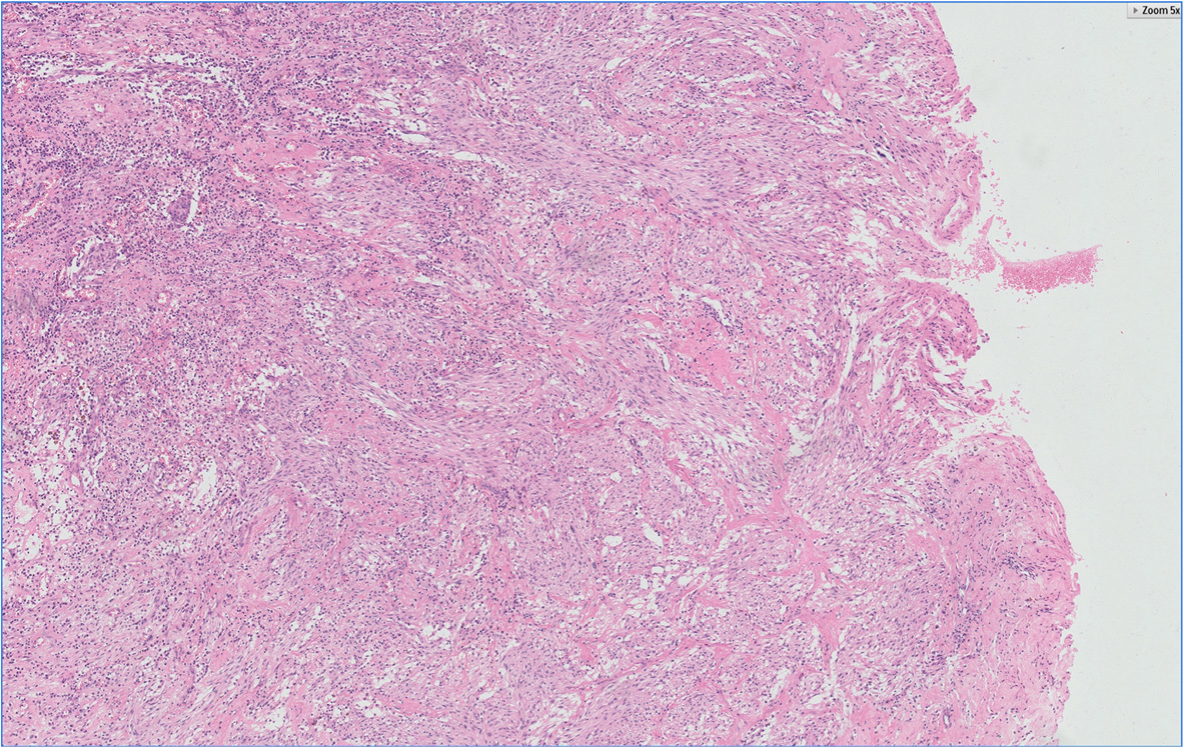
Histological images of the tumor with spindle cells and intersecting growth pattern Hemotoxylin eosin staining 5 ×.

**Figure 6:**
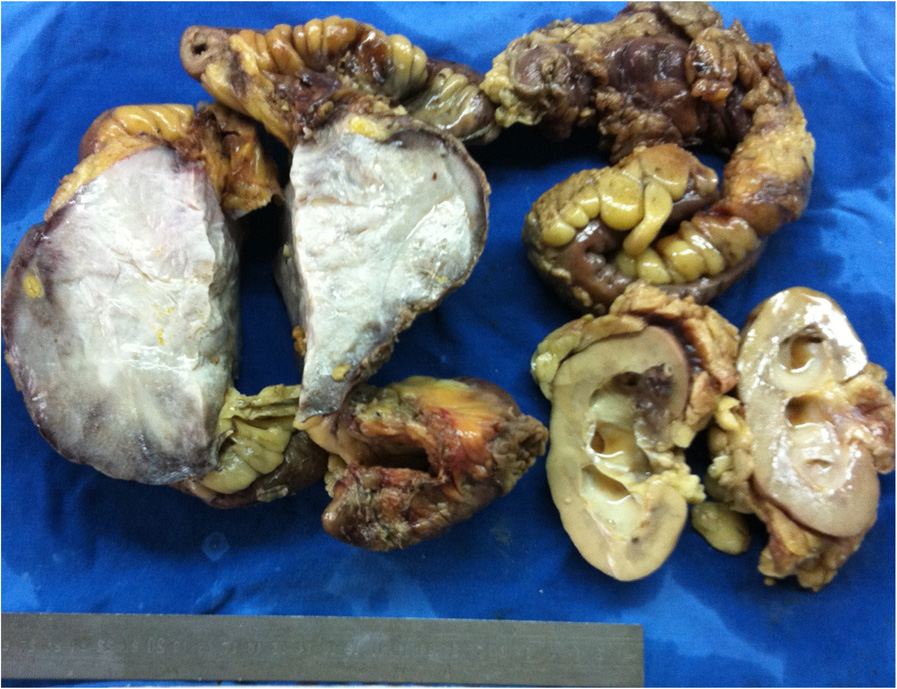
**Images of resected specimen, showing an intra-abdominal tumor with sacrifice of partial resection of the jejunum, colon, and left kidney in the small bowel mesentery and its surface section appearance, which is white and compact,and has no necrosis, hemorrhage or cystic changes.** The size of the tumor was 17 cm × 13 cm × 11 cm.

**Figure 7:**
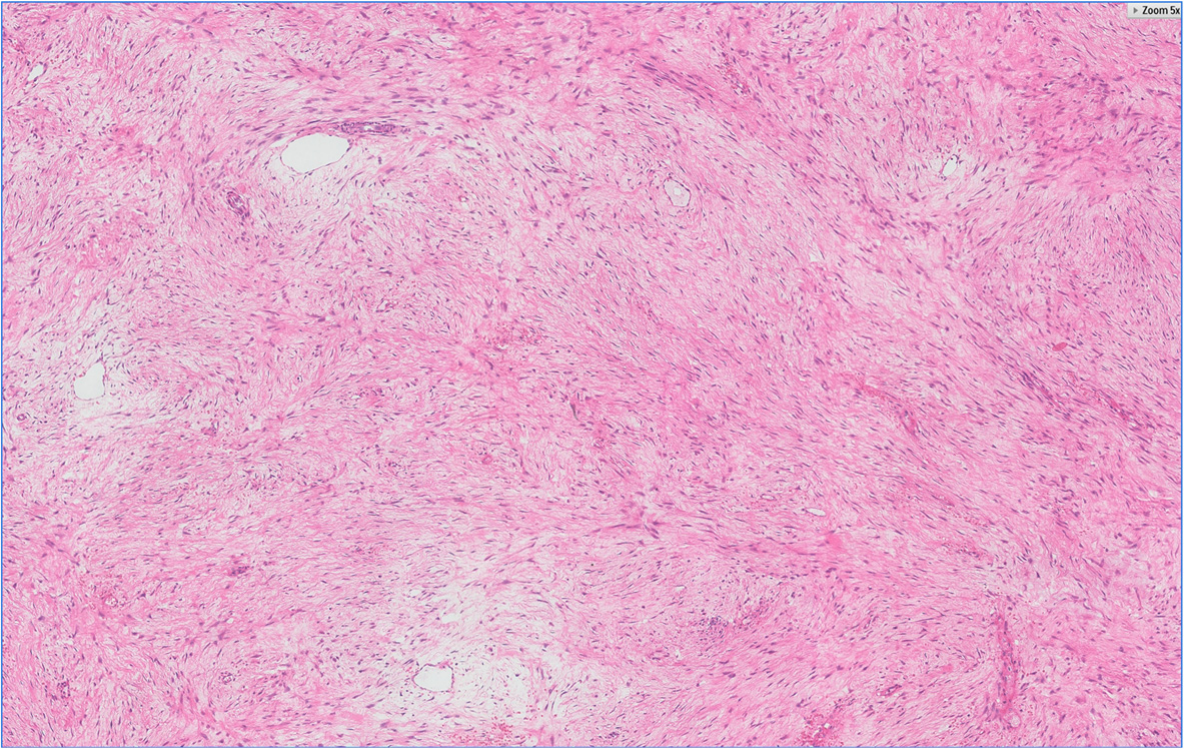
Microscopic images of the tumor (5×), showing loosely arranged spindle cells with bland, oval nuclei and minimal cytoplasm, plump spindle cells with tapering ends, oval, vesicular nuclei, moderate amounts of eosinophilic cytoplasm, and several thin-walled vessels of varying calibers.

Patient 2: A 45-year-old male was found to have duodenal tumor by physical examination in Aug 2008. He was treated with partial duodenal resection together with tumor on Aug 19th, 2008 in a local hospital (Haining County People’s Hospital, Zhejiang Province). The tumor was 6 cm × 4 cm × 3 cm in size. Microscopical examination found non-mitotic, spindle cells with intersecting growth pattern. The tumor cells were positive for CD117 and CD34, but negative for α-SMA, desmin and S-100 by immunohistochemistry. He was diagnosed as GIST and classified into moderate risk level. The patient was not treated with any adjuvant therapy after the operation. Twelve months later, a solitary and localized abdominal tumor with 4 cm in diameter was detected in the left upper abdominal cavity in routine CT scanning. The patient was reluctant to treatment. Six months later, the tumor was increased to 8 cm. He was initially treated with imatinib (400 mg daily) on Mar 28th, 2010 since the first clinical impression was recurrence of GIST. But CT scan performed 3 months after initiation of imatinib therapy found the tumor size was increased and CT scan performed 6 months after initiation of imatinib therapy revealed significant progression of the left abdominal tumor: its size enlarged to 15 cm. At this point, the patient was referred to our hospital for further management. A multidisciplinary team (MDT) meeting was convened. Physical examination identified a huge palpable mass in the left middle abdominal cavity. Abdominal ultrasonography revealed a homogeneous low-echoic tumor of 15 cm in diameter. CT scan revealed a low-dense, homogeneous tumor, mainly involving mesentery and mesenteric veins and adjacent to jejunum and colon, the ureter was also entrapped in it (Figures 3 and 4). Angiography showed a hypovascular tumor encroaching on superior mesenteric artery and vein, and their branches. Urography found the tumor obstructing the upper part of the left ureter, causing left side hydronephrosis. Furthermore, the primary duodenal GIST (Figure 5) was reevaluated as borderline nature and no *KIT* and *PDGFRA* mutation was found in the tumor. Therefore, a MDT decision was made to resect the lesion surgically. Prior to surgery, detailed examinations were performed. On Nov. 11th, 2010, the patient underwent exploratory laparotomy. The mesenteric tumor was successfully excised completely with adherent tissues and organs. Laparotomy revealed a hard tumor of 15 cm which appeared to originate from jejunal mesentery, involving proximal jejunum and the descending colon. The tumor also invaded and entrapped a segment of the upper part of ureter. The involved parts of jejunum and the tumor-bearing jejunal mesentery were resected en bloc along with the affected part of the mesocolon and the left kidney and the ureter. Grossly, the mass was resected with adequate margins and measured as 17 cm × 13 cm × 11cm. On section, the tumor was white and firm mass without necrosis, cystic change and hemorrhage (Figure 6). Microscopic examination of the tumor showed that non-dysplastic fibroblasts proliferating in the jejunal mesentery had infiltrated into the adjacent small intestine and ureter (Figure 7). The tumor cells were negative for CD117, S100, CD34, a-SMA and desmin. Therefore, the lesion was diagnosed as a mesenteric IAF. The postoperative course was uneventful without adjuvant therapy and no local recurrence has been noted as of October 2012.

## Discussion

Fibromatosis is a group of fibroblastic or myofibroblastic tissues that can be locally aggressive but do not metastasize [16]. The incidence of desmoids tumor has been reported as 2–4 cases per 1 million population, and deep fibromatosis, such as those arising in the abdominal cavity, are often referred to as IAF [17], which accounts for 8% of fibromatosis [18]. Although rare, IAF is the most common primary mesenteric tumor with spindle cell morphology [19]. Its causes and underlying mechanisms are unknown. Although most cases are sporadic, about 20% cases are associated with familial adenomatous polyposis (FAP) in a syndrome known as Gardner syndrome [20], about 10% cases with abdominal surgery or trauma experiences for various reasons [17,21-24], and rare cases with prior radiation therapy [25,26]. The two cases reported here were associated with previous abdominal surgery for GIST.

Of the above-mentioned situations, IAF developed after surgical resection for other tumors has special clinical significance. IAF has been observed in the sites of previous abdominal surgery for tumors [23,24]. Its diagnosis often is difficult to establish preoperatively, and it is usually misdiagnosed as recurrence at first clinical impression [2,22,23,27]. Surgical excision of the lesion is a difficult decision owing to the suspicion of metastasis mainly due to the following reasons. 1) The appearance of IAF on contrast enhanced imaging is not specific, therefore, the imaging diagnosis of IAF developed after abdominal surgery for other tumors is very difficult except in patients with familial adenomatous polyposis [20,28]. 2) The time interval between surgery and development of fibromatosis ranges from 2 months to several years (2.6 years on average) [17], which overlaps with recurrent disease.

Recently, there have been prior several case reports describing IAF arising on the site of a previously excised GIST. In these cases, IAF was first misdiagnosed as GIST recurrence [2,3]. Very recently, a non-random association between GIST and IAF was described. However, it’s different from a non-random association between GIST and myeloid leukemia [4], since an accurate diagnosis could only be established after surgical removal and pathological examination, as there are no typical imaging findings to suggest a IAF. Although it is a very rare event, IAF developed synchronously or metachronously with GIST could occur in most medical centers, for example, 28 IAF patients were collected from 10 medical centers [19].

Introduction of imatinib has greatly changed the clinical approach to intra-abdominal stromal spindle cell tumors. GIST is the most common mesenchymal tumor in gastrointestinal tract. Oncogenic mutations in *KIT* or *PDGFRA* have been identified as central tumor-initiating events in many GISTs [29]. Treatments with imatinib and sunitinib, two small-molecule inhibitors of the mutant *KIT* and *PDGFRA* receptor tyrosine kinases, significantly prolong survival of patients with GIST [1]. The median time for disease progression is 18–24 months in imatinib-treated patients with unresectable GIST. The clinical decision has been changed in patients with recurrent GIST.

In this study, the first patient had the disease in the era before imatinib. Since GIST is resistant to conventional cytotoxic chemotherapy [30], surgical resection was the first option for the patients. The second patient had the disease in the imatinib era, therefore, imatinib therapy was the first recommendation. However, the patient was not benefited from imatinib treatment: the tumor continued to grow rapidly after six months of imatinib therapy. After referred to our hospital, pathological re-evaluation suggested that the primary GIST was of borderline nature and the opportunity for recurrence was very low based on our previous experiences [31,32]. Furthermore, mutations in *KIT* and *PDGFRA* were not found in GIST. Since imatinib is less effective against GIST without *KIT* or *PDGFRA* mutation [33], and initial studies suggest that sunitinib treatment rarely results in objective responses in GIST [34,35], debulking surgery remains a recognized standard practice in the case of local progression because such procedure could prolong survival of patients who are resistant or insensitive to imatinib treatment [36]. Therefore, the decision of surgical resection was made for cure and definite diagnosis after MDT discussion.

Surgical intervention for IAF is generally considered to be the treatment of choice and is curative in many cases. Some studies have reported better prognosis for IAF patients with non-Gardner’s-associated IAF than for those complicated by Gardner’s syndrome [8,10,17,37]. In the study, both patients had no family history of FAP and uneventful prognosis. Complete surgical resection remains the cornerstone of management of IAF, while unresectable or residual disease can be treated with multiple choices. Nonsurgical treatment protocols mainly rely on sulindac [28], toremifene [38], cytotoxic chemotherapy [39], or in some circumstances, radiotherapy [40]. Each of them has variable and unpredictable efficacy [28]. Therefore. new treatment protocols for IAF are gradually being recommended, such as imatinib [41], sunitinib [42], bevacizumab [43], or sorafenib [44]. However, the two cases reported had been treated with imatinib at a dose of 400 mg for liver metastatic GIST. One achieved four years of long-term stable control [3] and the other achieved 11 months of disease control [2], but both developed IAF in primary GIST bed. The second patient was not benefit from imatinib therapy at this dosage.

Mace et al. recommended imatinib treatment at dose of 400 mg giving twice per day as a new therapeutic approach for desmoids tumor [45]. In Dumont’s cases, a patient who received imatinib at 400 mg/day for gastric GIST developed IAF on the posterior wall of the gastric antrum 35 months after initial diagnosis. After increasing imatinib dose to 800 mg/day, patient partially responded to both tumors [12], suggesting that patients with IAF might benefit from high dosage of imatinib.

Nevertheless, surgical trauma at the GIST excision site may predispose to the development of the IAF. This situation broads differential diagnosis and elicits a range of potential treatment options ranging from imatinib therapy to aggressive surgical re-excision for IAF. An accurate diagnosis is possible only after surgical removal and pathological examination, as there are no typical imaging findings to suggest IAF. Excluding diagnosis of recurrence of GIST is crucial for further management of our patients due to the increasing use of imatinib in the treatment of advanced GIST. In rare instances as illustrated in our cases, co-existence of another disease should be considered. The current two cases highlight the need for careful consideration of IAF when a rapidly growing spindle cell tumor is encountered in a post-GIST patient.

### Consent

Written informed consents were obtained from patients and their family members for publication of this report.

## Competing interests

The authors declare that they have no competing interests.

## Authors’ contributions

All authors read and approved the final manuscript.
